# Spatiotemporal Airy Ince–Gaussian wave packets in strongly nonlocal nonlinear media

**DOI:** 10.1038/s41598-018-22510-7

**Published:** 2018-03-08

**Authors:** Xi Peng, Jingli Zhuang, Yulian Peng, DongDong Li, Liping Zhang, Xingyu Chen, Fang Zhao, Dongmei Deng

**Affiliations:** Guangdong Provincial Key Laboratory of Nanophotonic Functional Materials and Devices, South China Normal University, Guangzhou, 510631 China

## Abstract

The self-accelerating Airy Ince–Gaussian (AiIG) and Airy helical Ince–Gaussian (AihIG) wave packets in strongly nonlocal nonlinear media (SNNM) are obtained by solving the strongly nonlocal nonlinear Schrödinger equation. For the first time, the propagation properties of three dimensional localized AiIG and AihIG breathers and solitons in the SNNM are demonstrated, these spatiotemporal wave packets maintain the self-accelerating and approximately non-dispersion properties in temporal dimension, periodically oscillating (breather state) or steady (soliton state) in spatial dimension. In particular, their numerical experiments of spatial intensity distribution, numerical simulations of spatiotemporal distribution, as well as the transverse energy flow and the angular momentum in SNNM are presented. Typical examples of the obtained solutions are based on the ratio between the input power and the critical power, the ellipticity and the strong nonlocality parameter. The comparisons of analytical solutions with numerical simulations and numerical experiments of the AiIG and AihIG optical solitons show that the numerical results agree well with the analytical solutions in the case of strong nonlocality.

## Introduction

Ince-Gaussian (IG) beams, which constitute the third complete family of transverse eigenmodes of stable resonators, have attracted extensive attention from research communities all over the world since it was introduced by Bandres and Gutiérrez-Vega^1,2^. The transverse structure of IG modes is described by the Ince polynomials, and the limiting cases of IG modes are Laguerre Gaussian (LG) modes and Hermite Gaussian (HG) modes when the ellipticity parameter of IG modes tends to zero or infinity, respectively. Meanwhile, Schwarz *et al*. have generated single high order IG modes with very high quality by slightly breaking the symmetry of the cavity of a diode pumped Nd:YVO_4_ laser and its pump beam configuration^3,4^.

Soon afterwards, Deng *et al*. have discovered the IG solitons in strongly nonlocal nonlinear media (SNNM)^5–7^. Recent experimental results demonstrate that nematic liquid crystals^8^ and lead glass^9^ are strongly nonlocal nonlinear media. Propagation properties of Airy beams in the SNNM has been studied by Zhou *et al*.^10^. Then, the anomalous interaction of Airy beams in nonlocal nonlinear media have been reported by Shen *et al*.^11^, in which nonlocal nonlinearity also affects the interaction of out-of-phase bright solitons and dark solitons. In addition, owing to the SNNM, Zhu *et al*. have obtained that the Airy vortex beams follow a periodic trajectory when propagating through a SNNM^12^.

Over the past decades, tremendous efforts have been made by researchers to generate the three-dimensional (3D) localized optical solitons^13–21^, which also called spatiotemporal light bullets, are localized in both space and time. Among which, Airy wave packets were widely reported in free space^16–18^ and quadratic index medium^20,21^ due to the self-accelerating^22–26^, self healing^27^ and no diffraction^28^ features of Airy distribution. It is worth mentioning some pioneer works in Airy related wave packets. The spatiotemporal Airy-Bessel light bullets by combining an Airy pulse with a two-dimensional Bessel beam have been studied by Chong *et al*.^14^. At the same time, Abdollahpour *et al*. have reported the spatiotemporal *Airy*^3^ light bullets by combining an Airy pulse in time with a spatial Airy beam^15^. Recent interest in the study of Airy related wave packets have undergone rapid development. For instance, in free space, Zhong *et al*. have reported the 3D localized Airy-Laguerre-Gaussian wave packets^16^, Deng *et al*. have studied the 3D localized Airy-Hermite-Gaussian and Airy-Helical-Hermite-Gaussian wave packets^17^, and Peng *et al*. have obtained the 3D localized Airy-Ince-Gaussian and Airy-Helical-Ince-Gaussian wave packets^18^. Then, the chirped Airy Gaussian vortex wave packets in quadratic index medium^20^ and Airy Gaussian and Airy Gaussian vortex light bullets in harmonic potential^21^ have been reported. However, what will happen when the 3D localized Airy Ince-Gaussian (AiIG) wave packets propagate in the SNNM? It will be significant to investigate the AiIG and Airy helical Ince-Gaussian (AihIG) optical breathers and solitons and their propagation dynamics in the SNNM.

## Methods

In the paraxial approximation and in the nonlocal nonlinear media, the propagation of the wave packet obeys the (1 + 3)D nonlocal nonlinear Schrödinger equation^5–7,29,30^

1 $$i\frac{{\rm{\partial }}U}{{\rm{\partial }}z}+\frac{1}{2k}(\frac{{{\rm{\partial }}}^{2}U}{{\rm{\partial }}{x}^{2}}+\frac{{{\rm{\partial }}}^{2}U}{{\rm{\partial }}{y}^{2}})-\frac{{k^{\prime\prime} }_{0}}{2}\frac{{{\rm{\partial }}}^{2}U}{{\rm{\partial }}{t}^{2}}+k\frac{{\rm{\Delta }}n}{{n}_{0}}U=0,$$

where *U*(*r*, *t*, *z*) = *U*(*x*, *y*, *t*, *z*) is a paraxial wave packet, *r* = $\sqrt{{x}^{2}+{y}^{2}}$, *x* and *y* are the transverse coordinates, *z* is the longitudinal coordinate (the propagation distance in nonlocal nonlinear materials), *k* is the wave number in the media without nonlinearity, *k*_0_′′ is the dispersion coefficient of the media evaluated at ω_0_. Δ*n* = *n*_2_∫*N*(*r* − *r*′)*|U*(*r*′, *z*)|^2^*d*^2^*r*′ is the spatial nonlinear perturbation of the refraction index, *n*_2_ is the nonlinear index coefficient, *r* and *r*′ are two-dimensional transverse coordinates, *N* is the normalized symmetrical real spatial response function of the media. Without loss of generality, we assume the material response to be the Gaussian function $N(r)=\exp [-{r}^{2}\mathrm{/(2}{w}_{m}^{2}\mathrm{)]/(2}\pi {w}_{m}^{2})$, where *w*_*m*_ is the characteristic length of the material response function.

Underlying the dimensionless coordinates (*X, Y, T, Z*) = (*x*/*w*_0_, *y*/*w*_0_, *t*/*t*_0_, *z*/*L*), w_0_ is the spatial scaling parameter, *t*_0_ is the temporal scaling parameter, $L=k{w}_{0}^{2}$ is the diffraction length. In the case of the strong nonlocality^29^, Eq. (1) can be simplified into the normalized dimensionless Snyder–Mitchell model

2 $$i\frac{\partial U}{\partial Z}+\frac{1}{2}(\frac{{\partial }^{2}U}{\partial {X}^{2}}+\frac{{\partial }^{2}U}{\partial {Y}^{2}}+\frac{{\partial }^{2}U}{\partial {T}^{2}})-\frac{{P}_{0}}{2{P}_{c}}({X}^{2}+{Y}^{2})U=\mathrm{0,}$$

where *P*_0_ is the input power at *Z* = 0, *P*_*c*_ = *n*_0_/(*γn*_2_*L*^2^) is the critical power for the soliton propagation, *γ* is the material parameter relating to *N*. Eq. (2) is an equation in the case of the nonlinearity limit with the degrees of nonlocality approaching to infinity, the field can change the refractive index of the medium while propagating, which is creating a structure similar to a graded-index fiber. Schrödinger equation in ref.^20^. is from the quadratic index medium, while Eq. (2) is from strongly nonlocal nonlinear medium condition, they are two different questions. The solution of Eq. (2) can be obtained from the method of separation of variables as

3 $$U(X,Y,T,Z)=H(\xi )F(J){e}^{iM(Z)}{{\varphi }}_{G}(X,Y,Z)A(T,Z),$$

in the transverse plane perpendicular to *Z*, the elliptic coordinates^1–5^ are defined as *X* = *f*(*Z*)*coshξcosJ*, *Y* = *f*(*Z*)*sinhξsinJ*, Z = Z, where $\xi \,\geqslant \,0$ and $0\,\leqslant \,J < 2\pi$ denote the radial and angular elliptic variables, respectively. Semifocal separation *f*(*Z*) = *f*_0_*w*(*Z*)/*w*_0_, *f*_0_ denotes semifocal separation at waist plane Z = 0. ξ and *J* satisfy continuity in the whole space. *ϕ*_*G*_ is the Gaussian solution^5,18^

4 $${{\varphi }}_{G}(X,Y,Z)=\frac{D}{w(Z)}{e}^{-\frac{{X}^{2}+{Y}^{2}}{{w}^{2}(Z)}+i\frac{{X}^{2}+{Y}^{2}}{2R(Z)}-i\arctan [\sqrt{\frac{{P}_{c}}{{P}_{0}}}tan(\sqrt{\frac{{P}_{0}}{{P}_{c}}}Z)]},$$

5 $$w(Z)={[{\cos }^{2}(\sqrt{\frac{{P}_{0}}{{P}_{c}}}Z)+\frac{{P}_{c}}{{P}_{0}}{\sin }^{2}(\sqrt{\frac{{P}_{0}}{{P}_{c}}}Z)]}^{\mathrm{1/2}},$$

6 $$R(Z)=\sqrt{\frac{{P}_{0}}{{P}_{c}}}[\tan (\sqrt{\frac{{P}_{0}}{{P}_{c}}}Z)+\frac{{P}_{0}}{{P}_{c}}\,\cot (\sqrt{\frac{{P}_{0}}{{P}_{c}}}Z)]/(1-\frac{{P}_{0}}{{P}_{c}}),$$

where D is a normalization constant.

By substituting *U*(*X*, *Y*, *T*, *Z*) into Eq. (2), we have the following equations

7 $$i\frac{\partial A}{\partial Z}+\frac{1}{2}\frac{{\partial }^{2}A}{\partial {T}^{2}}=\mathrm{0,}$$

8 $$-{f}^{2}\frac{dM}{dZ}=\varepsilon p,$$

9 $$\frac{{\partial }^{2}H}{\partial {\xi }^{2}}-\varepsilon \,\sinh \,2\xi \frac{\partial H}{\partial \xi }-(a-p\varepsilon \,\cosh \,2\xi )H=\mathrm{0,}$$

10 $$\frac{{\partial }^{2}F}{\partial {J}^{2}}+\varepsilon \,\sin \,2J\frac{\partial F}{\partial J}+(a-p\varepsilon \,\cos \,2J)F=\mathrm{0,}$$

where *a* and *p* are separation constants and $\varepsilon =2{f}_{0}^{2}/{w}_{0}^{2}$ is the ellipticity parameter of the IG wave packet.

In Eq. (7), we deal with finite energy Airy wave packet for the physical reality, which can be expressed as *A*(*T*, 0) = *Ai*(*T*)*exp*(*σT*), where *Ai*(*T*) is the Airy function^22–24^, $\sigma \mathrm{(0} < \sigma \,\leqslant \,\mathrm{1)}$ is the decay factor. By using the Fourier-transform method, one can obtain the solution of Eq. (7) as

11 $$A(T,Z)=Ai(T-\frac{{Z}^{2}}{4}+i\sigma Z){e}^{\sigma T-\frac{1}{2}\sigma {Z}^{2}+i(-\frac{1}{12}{Z}^{3}+\frac{1}{2}{\sigma }^{2}Z+\frac{1}{2}TZ)}\mathrm{.}$$

The solution of Eq. (8) can be given as

12 $$M(Z)=-p\,\arctan [\sqrt{{P}_{c}/{P}_{0}}\,\tan (Z\sqrt{{P}_{0}/{P}_{c}}\mathrm{)].}$$

Equation (10) is the Ince equation^1–7^, which is a special case of the Hill equation. If *iξ* = *J*, Eq. (9) can be transformed into Eq. (10), and vice versa. The solutions of Eq. (10) are known as the even and odd Ince polynomials of order *p* and degree *m*, usually expressed as ${{C}}_{p}^{m}$ and ${S}_{p}^{m}$^1–3^, where $0\,\leqslant \,m\,\leqslant \,p$ for an even function, $1\,\leqslant \,m\,\leqslant \,p$ for an odd function, and indices (*p*, *m*) have the same parity. Then, the spatial even IG, odd IG and helical IG (hIG) distribution can be expressed as

13 $$I{G}^{e}(X,Y,Z)={H}^{e}(\xi ){F}^{e}(J){e}^{iM(Z)}{{\varphi }}_{G}(X,Y,Z)=\frac{D}{w(Z)}{C}_{p}^{m}(i\xi ,\varepsilon ){C}_{p}^{m}(J,\varepsilon ){e}^{-\frac{{X}^{2}+{Y}^{2}}{{w}^{2}(Z)}+i[\delta Z+\frac{{X}^{2}+{Y}^{2}}{2R(Z)}-\theta (Z)]},$$

14 $$I{G}^{o}(X,Y,Z)={H}^{o}(\xi ){F}^{o}(J){e}^{iM(Z)}{{\varphi }}_{G}(X,Y,Z)=\frac{D}{w(Z)}{S}_{p}^{m}(i\xi ,\varepsilon ){S}_{p}^{m}(J,\varepsilon ){e}^{-\frac{{X}^{2}+{Y}^{2}}{{w}^{2}(Z)}+i[\delta Z+\frac{{X}^{2}+{Y}^{2}}{2R(Z)}-\theta (Z)]},$$

15 $$I{G}^{h}(X,Y,Z)=I{G}^{e}+iI{G}^{o}=\frac{D}{w(Z)}[{C}_{p}^{m}(i\xi ,\varepsilon ){C}_{p}^{m}(J,\varepsilon )+i{S}_{p}^{m}(i\xi ,\varepsilon ){S}_{p}^{m}(J,\varepsilon )]{e}^{-\frac{{X}^{2}+{Y}^{2}}{{w}^{2}(Z)}+i[\delta Z+\frac{{X}^{2}+{Y}^{2}}{2R(Z)}-\theta (Z)]},$$

where $\delta ={k}^{2}{w}_{0}^{2}$, *θ*(*Z*) = (*p* + 1)arctan[$\sqrt{{P}_{c}/{P}_{0}}$tan(Z$\sqrt{{P}_{0}/{P}_{c}}$)].

The even AiIG, odd AiIG and AihIG wave packets in SNNM can be expressed as

16 $$\begin{array}{rcl}{U}^{e}(X,Y,T,Z)=A(T,Z)I{G}^{e}(X,Y,Z) & = & \frac{D}{w(Z)}{C}_{p}^{m}(i\xi ,\varepsilon ){C}_{p}^{m}(J,\varepsilon ){e}^{-\frac{{X}^{2}+{Y}^{2}}{{w}^{2}(Z)}+i[\delta Z+\frac{{X}^{2}+{Y}^{2}}{2R(Z)}-\theta (Z)]}\\  &  & \times \,Ai(T-\frac{{Z}^{2}}{4}+i\sigma Z){e}^{\sigma T-\frac{1}{2}\sigma {Z}^{2}+i(-\frac{1}{12}{Z}^{3}+\frac{1}{2}{\sigma }^{2}Z+\frac{1}{2}TZ)},\end{array}$$

17 $$\begin{array}{rcl}{U}^{o}(X,Y,T,Z)=A(T,Z)I{G}^{o}(X,Y,Z) & = & \frac{D}{w(Z)}{S}_{p}^{m}(i\xi ,\varepsilon ){S}_{p}^{m}(J,\varepsilon ){e}^{-\frac{{X}^{2}+{Y}^{2}}{{w}^{2}(Z)}+i[\delta Z+\frac{{X}^{2}+{Y}^{2}}{2R(Z)}-\theta (Z)]}\\  &  & \times \,Ai(T-\frac{{Z}^{2}}{4}+i\sigma Z){e}^{\sigma T-\frac{1}{2}\sigma {Z}^{2}+i(-\frac{1}{12}{Z}^{3}+\frac{1}{2}{\sigma }^{2}Z+\frac{1}{2}TZ)},\end{array}$$

18 $$\begin{array}{rcl}{U}^{h}(X,Y,T,Z) & = & {U}^{e}(X,Y,T,Z)+i{U}^{o}(X,Y,T,Z)\\  & = & \frac{D}{w(Z)}[{C}_{p}^{m}(i\xi ,\varepsilon ){C}_{p}^{m}(J,\varepsilon )+i{S}_{p}^{m}(i\xi ,\varepsilon ){S}_{p}^{m}(J,\varepsilon )]{e}^{-\frac{{X}^{2}+{Y}^{2}}{{w}^{2}(Z)}+i[\delta Z+\frac{{X}^{2}+{Y}^{2}}{2R(Z)}-\theta (Z)]}\\  &  & \times \,Ai(T-\frac{{Z}^{2}}{4}+i\sigma Z){e}^{\sigma T-\frac{1}{2}\sigma {Z}^{2}+i(-\frac{1}{12}{Z}^{3}+\frac{1}{2}{\sigma }^{2}Z+\frac{1}{2}TZ)}\mathrm{.}\end{array}$$

## Results and Discussion

Having found the analytical solutions of Eqs (16–18) in SNNM, we concentrate on the comparison of analytical solutions with numerical simulations and numerical experiments of the spatiotemporal AiIG and AihIG wave packets in the following. We discuss various examples of the localized wave packets for different parameters. The evolution properties of the AiIG and AihIG wave packets can be easily manipulated and modulated through adjusting the ratio between the input power and the critical power, the ellipticity and the strong nonlocality parameter.

**Figure 1:**
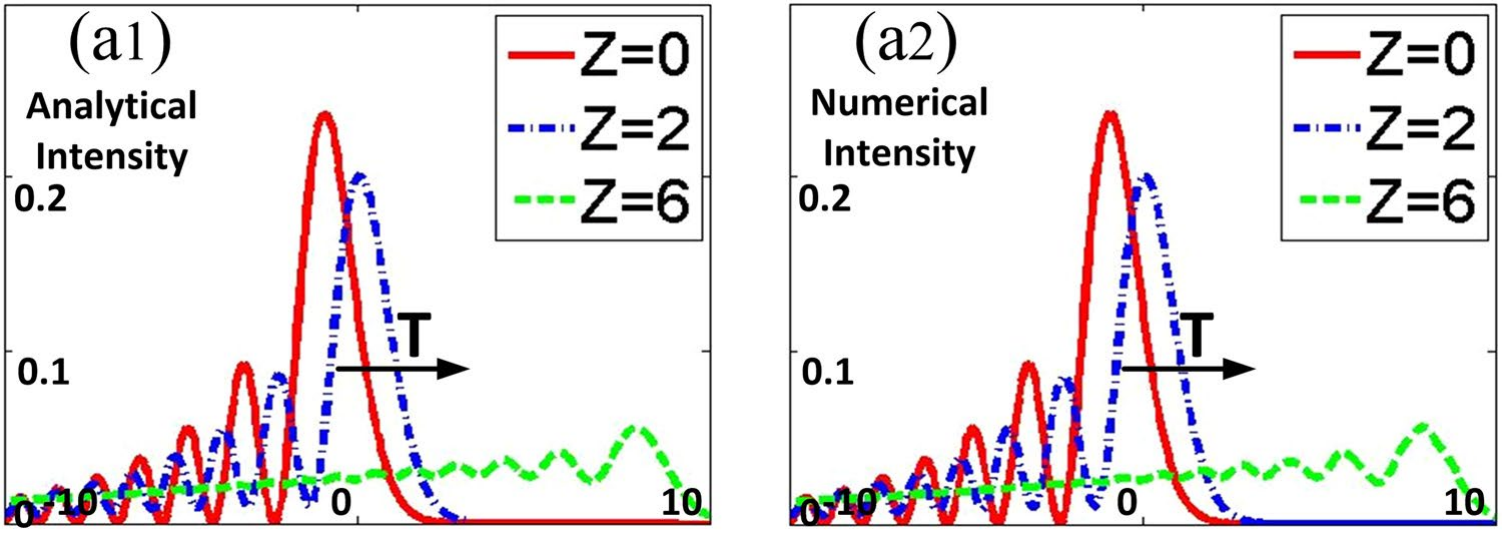
(**a1**) The analytical intensity profiles [*I* = |*A*(*T*, *Z*)|^2^] of finite-energy Airy pulses at various propagation distances *Z*, (**a2**) the numerical intensity profiles of Eq. (7) by using the split step fourier transform.

**Figure 2:**
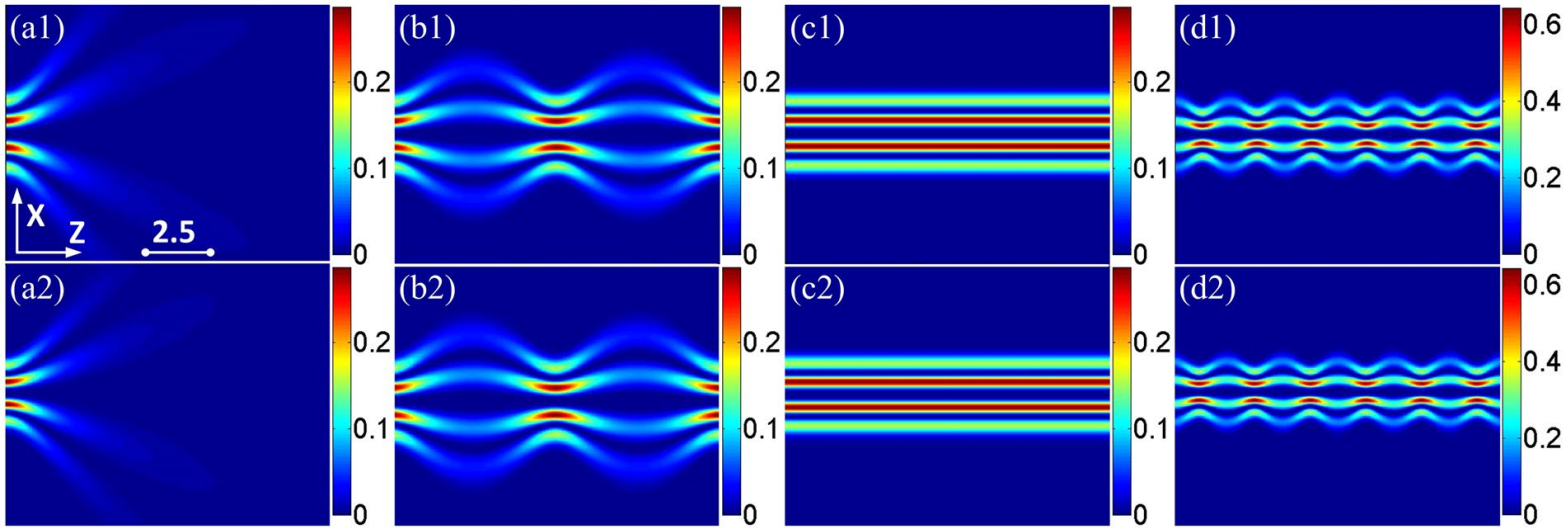
(**a1**–**d1**) The analytical propagation of the spatial IG wave packets *IG*^*o*^(*X*, *Y* = *X*, *Z*) with *ε* = 3, p = m = 4. (**a1**) *P*_0_/*P*_*c*_ = 10^−3^, (**b1**) *P*_0_/*P*_*c*_ = 0.5, (**c1**) *P*_0_/*P*_*c*_ = 1, and (**d1**) *P*_0_/*P*_*c*_ = 1.5. (**a2**–**d2**) The related numerical simulations [spatial part of Eq. (2)].

By comparing Fig. 1(a1) with Fig. 1(a2), there is very good qualitative agreement between analytical result and numerical simulation of the temporal Airy wave packets. Propagation of the spatial odd IG wave packets is shown in Fig. 2(a1–d1), for different choices of the parameter *P*_0_/*P*_*c*_. Without doubt, the wave is free to broaden when *P*_0_ approaches to zero. IG breathers initially broaden because wave packet diffraction initially overcomes wave packet induced refraction as *P*_0_ = 0.5*P*_*c*_ in Fig. 2(b1). The comparison shows that the IG solitons propagate stably with the input power equaling the critical power in Fig. 2(c1), which means that diffraction is exactly balanced by nonlinearity. And the IG breathers initially narrow as *P*_0_ = 1.5*P*_*c*_ in Fig. 2(d1). The numerical simulation of the spatial propagation distributions is shown in Fig. 2(a2–d2), which agrees well with the related analytical result. The spatiotemporal AiIG solitons can be realised theoretically when the input power equals the critical power, with steady state in spatial and almost non-dispersion in temporal dimensions. On the other hand, we can achieve 3D localized AiIG breathers periodically oscillating when the balance between diffraction and nonlinearity is broken.

**Figure 3:**
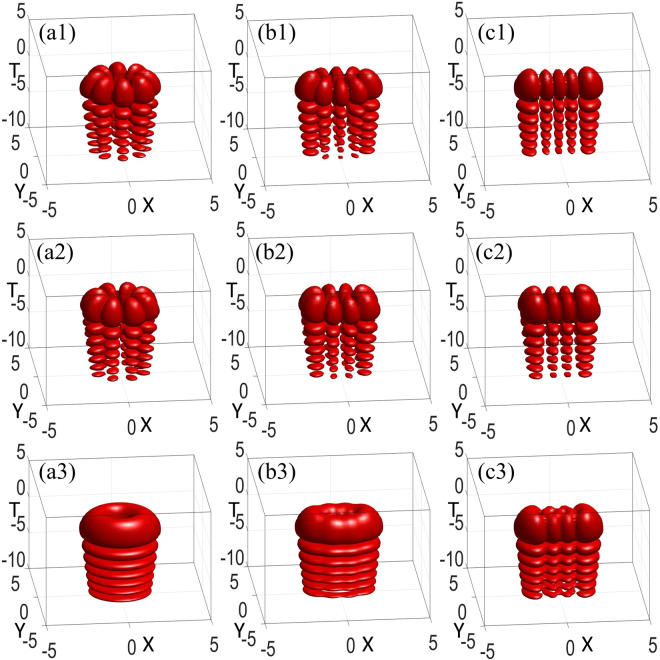
Snapshots describing the initial spatiotemporal even (**a1**–**c1**), odd (**a2**–**c2**), and helical (**a3**–**c3**) AiIG wave packets *AiIG*(*X*, *Y*, *T*, *Z*) with (**a1**–**a3**) *ε* = 10^−5^, (**b1**–**b3**) *ε* = 3, and (**c1**–**c3**) *ε* = 10^5^. *P*_0_ = *P*_*c*_, *p* = *m* = 4, *Z* = 0.

**Figure 4:**
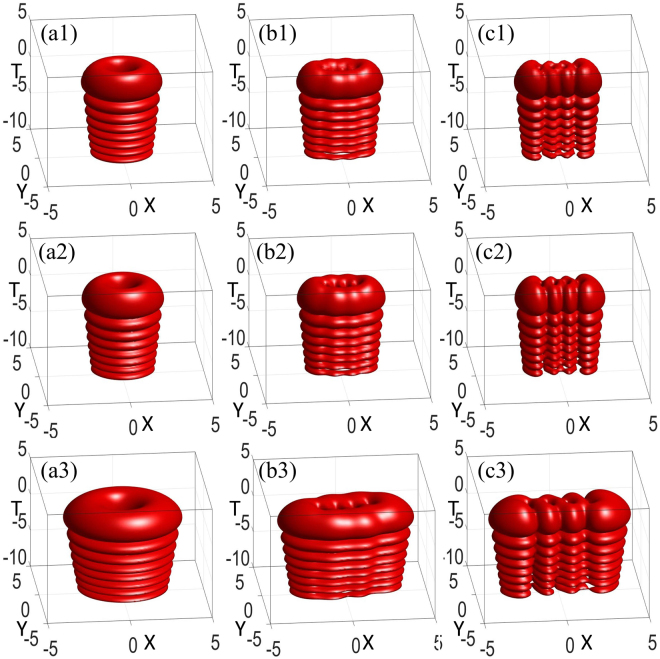
(**a1**–**c1**) Snapshots describing the analytical solution of AihIG wave packets, the parameters are the same as those in Fig. 2(a3–c3) except *Z* = 2. Snapshots describing the numerical simulation [Eq. (2)] by using the split step Fourier transform with (**a2**–**c2**) *α* = 0.01, (**a3**–**c3**) *α* = 0.5, at *Z* = 2.

As revealed, the initial spatiotemporal AiIG and AihIG soliton patterns vary with the ellipticity ε are shown in Fig. 3. Under the condition of the input power equaling the critical power, we obtain an approach to generate approximately steady 3D AihIG solitons, which can maintain the shape after propagating many Rayleigh lengths by compare Fig. 4(a1–c1) with Fig. 3(a3–c3). Actually, the peaks along the *T* direction go ahead after propagation due to the self-accelerating character of Airy wave packets, which can also be found by comparing different propagation distances in Fig. 1(a1) and (a2). In addition, we utilize split step Fourier transform to show the numerical simulation, *α* = *w*_0_/*w*_*m*_ denotes the degree of the material nonlocality. The less *α* is, the stronger the nonlocality is. The numerical results (Fig. 4(a2–c2)) agree well with the analytical solutions (Fig. 4(a1–c1)) in the case of strong nonlocality with *α* = 0.01. However, at the same propagation distance, the numerical simulation results present unstable properties when the degree of nonlocality becomes weaker (*α* = 0.5), as shown in Fig. 4(a3–c3).

Likewise, one can easily obtain the self-accelerating AiIG breathers periodically oscillating when the balance between diffraction and nonlinearity is broken. The phenomenon shows the spatial periodically oscillating (breather state) or steady (soliton state), temporal self-accelerating and approximately non-dispersion properties of AiIG breathers and solitons reported in SNNM are different from other spatiotemporal Airy related wave packets diffracting in spatial dimension while propagating in free space^17,18^. Without loss of generality, the relationship of AiIG, Airy Laguerre Gaussian (AiLG), and Airy Hermite Gaussian (AiHG) breathers and solitons will be analysed as follow. When *ε* → 0, the transition from *IG*_*pm*_ to *LG*_*nl*_ occurs. Simultaneously, the indices of both modes are related as: *l = m* and *n* = (*p* − *m*)/2 in the limit. When *ε* → ∞, the transition from *IG*_*pm*_ to $H{G}_{{l}_{x}{l}_{y}}$ occurs. In the limit, the indices are related as: for even AiIG breathers and solitons *l*_*x*_ *= m* and *l*_*y*_ *= p* − *m*, on the contrary, for odd AiIG breathers and solitons *l*_*x*_ *= m* − 1 and *l*_*y*_ *= p* − *m* + 1.

**Figure 5:**
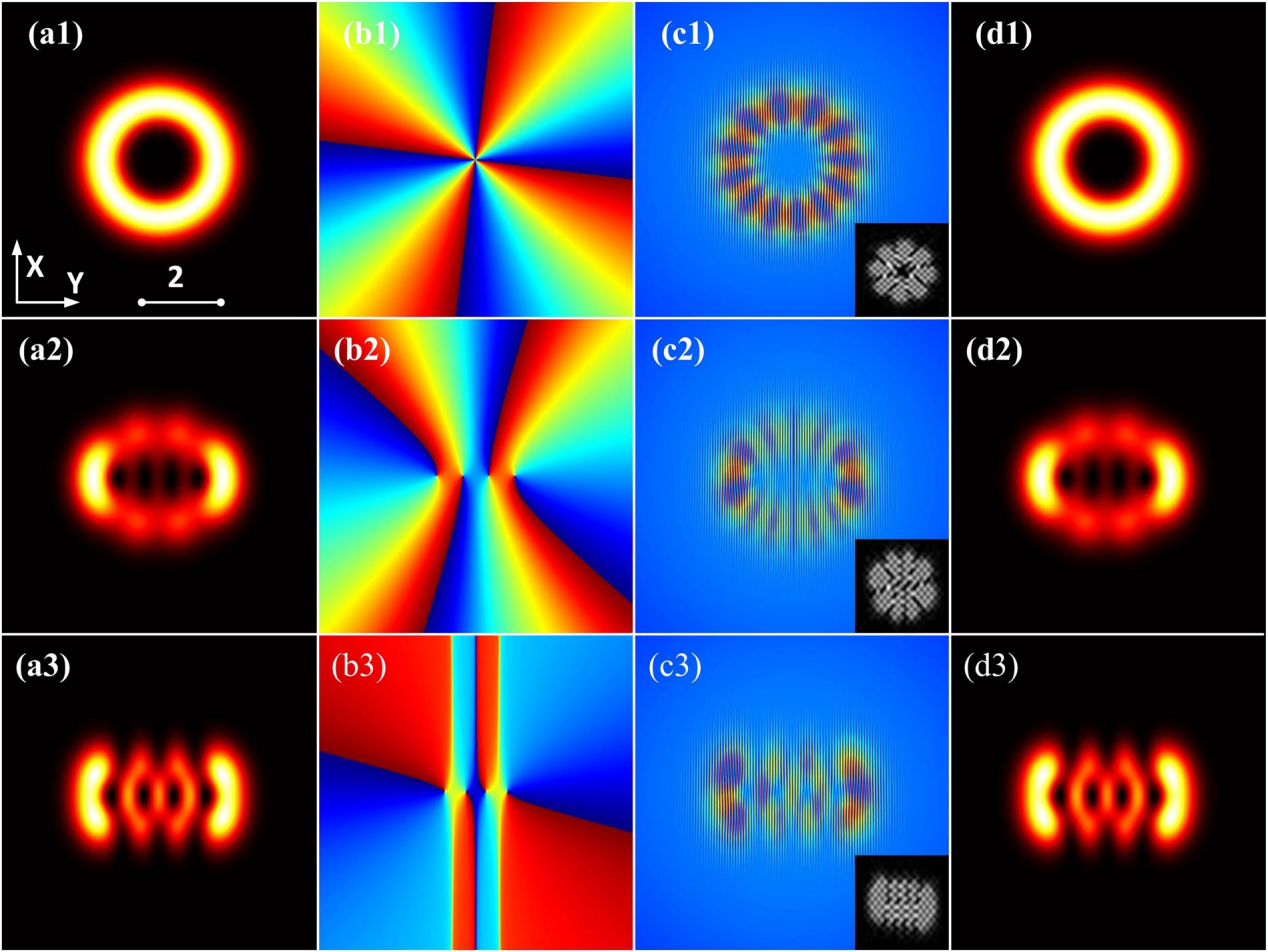
(**a1**–**a3**) Analytical intensity distributions of the hIG wave packet evolution. (**b1**–**b3**) Phase distributions. (**c1**–**c3**) Interference intensity of the initial generated wave packet and a plane wave, the gray insets display the related computer-generated hologram. (**d1**–**d3**) Numerical experimentally [spatial part of Eq. (2)] recorded the transverse intensity distributions at *Z* = 2, with (**a1**–**d1**) *ε* = 10^−5^, (**a2**–**d2**) *ε* = 3, and (**a3**–**d3**) *ε* = 10^5^. *P*_0_ = *P*_*c*_.

**Figure 6:**
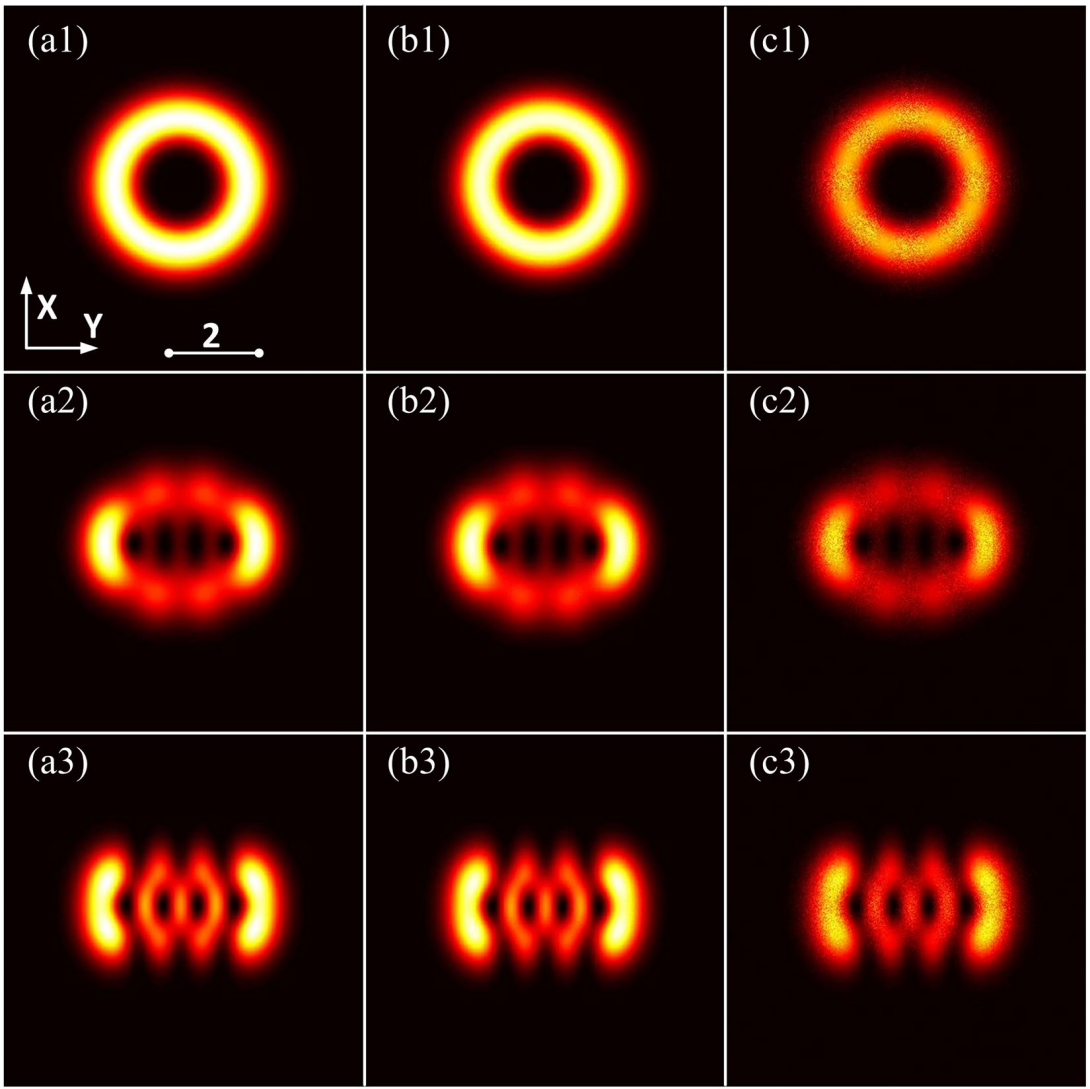
Numerical simulations [spatial part of Eq. (2)] of the intensity distributions of hIG wave packets in SNNM with initial perturbation. (**a1**–**a3**) *ρ* = 0 (soliton state), (**b1**–**b3**) *ρ* = 0.01, and (**c1**–**c3**) *ρ* = 0.05. (**a1**–**c1**) *ε* = 10^−5^, (**a2**–**c2**) *ε* = 3, and (**a3**–**c3**) *ε* = 10^5^. *P*_0_ = *P*_*c*_, *Z* = 3.

We formulate the spatial hIG wave packet analytically in SNNM and present related numerical experiment results of the hIG wave packet generation, corroborating the properties we describe. The intensity patterns of the initial input of hIG beams are shown in Fig. 5(a1–a3). The phase patterns of the initial input of hIG beams are shown in Fig. 5(b1–b3), where the locations of the optical vortexes are obtained. For numerical experimental generation, we launch an initial beam [see Fig. 5(a1–a3)] to reconstruct the computer-generated holograms [see gray insets display of Fig. 5(c1–c3)] of the desired beam profiles^18^. The holograms are obtained by computing the interference patterns between the complex amplitude profile of the hIG beams at the *Z* = 0 plane [see Fig. 5(a1–a3)] and a plane wave [see Fig. 5(c1–c3)]. In the frequency domain, the information of the first order interference is chosen to produce the new wave packets. The transverse intensity patterns taken at *Z* = 2 [see Fig. 5(d1–d3)] indicate clearly that the hIG beams maintain the same along the propagation. By comparing Fig. 5(a1–a3) (analytical results) and Fig. 5(d1–d3) (numerical experiments) with Fig. 6(a1–a3) (numerical simulations), our results show a good agreement among them.

**Figure 7:**
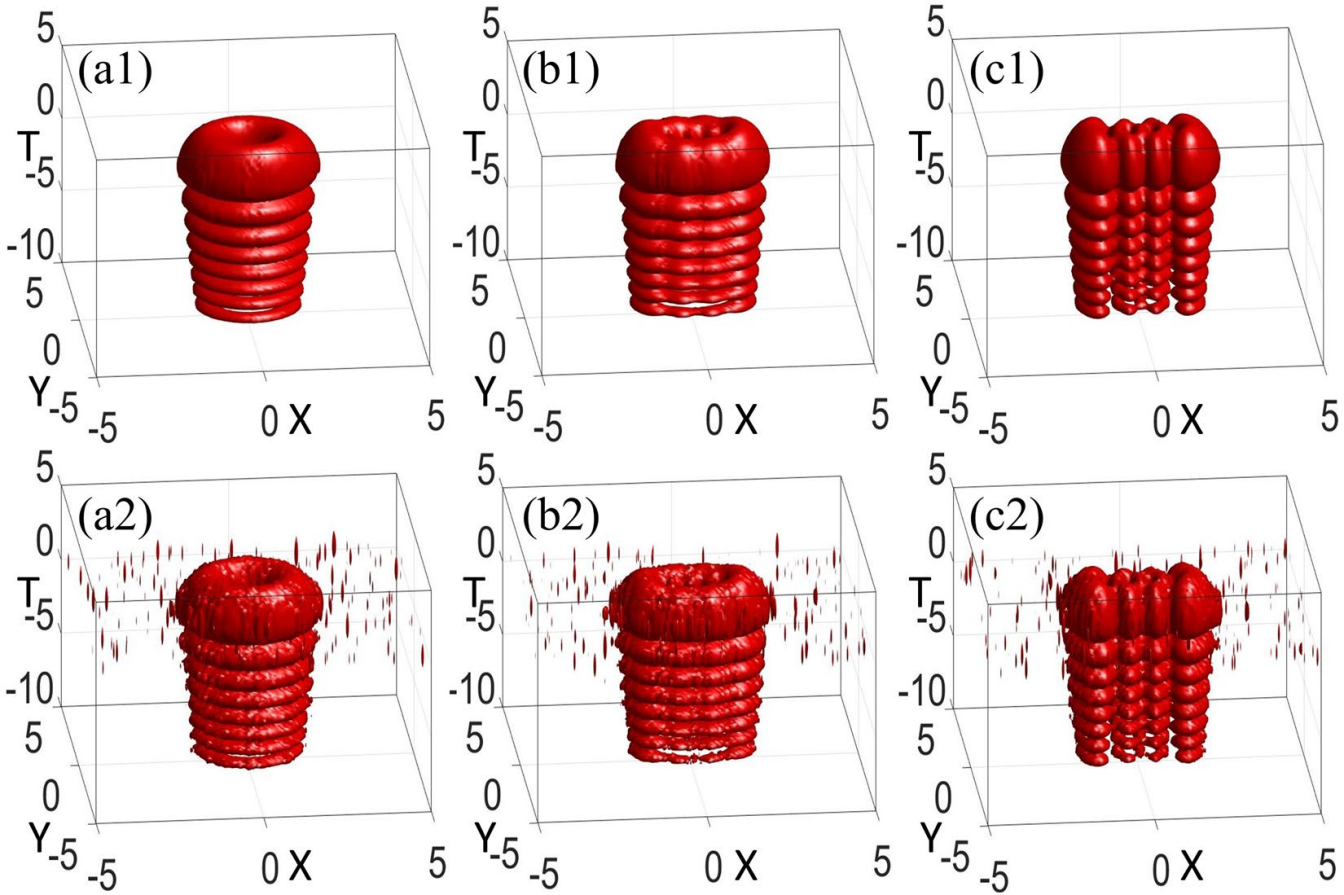
Snapshots describing the numerical simulation [Eq. (2)] of AihIG wave packets. (**a1**–**c1**) *ρ* = 0.01, (**a2**–**c2**) *ρ* = 0.05. (**a1**–**a2**) *ε* = 10^−5^, (**b1**–**b2**) *ε* = 3, (**c1**–**c2**) *ε* = 10^5^. *P*_0_ = *P*_*c*_, *Z* = 3.

To obtain the stability analysis of the hIG wave packets, in Fig. 6, we numerically simulate the intensity distribution, which is excited by an initial perturbation^31^. The initial condition is supposed to be *IG*^*h*^(*X*, *Y*, *Z* = 0) + *ρψ*(*X*, *Y*, *Z* = 0), where *ψ*(*X*, *Y*, 0) is a random complex function whose maximum amplitude is less than that of the hIG beam, and *ρ* denotes the perturbation parameter. The difference between the output intensity distribution of the hIG beams and the solitons is huge when *ρ* = 0.05 in Fig. 6(c1–c3); the difference becomes so small that one can hardly distinguish it when *ρ* = 0.01 in Fig. 6(b1–b3). Stable soliton propagation in a numerical experiment where the initial beam is perturbed by noise, which may not constitute a rigorous proof of the stability, but does provide strong support for the existence of observable nonlinear modes in laboratory experiments. The related numerical simulations of spatiotemporal AihIG wave packets with *ρ* = 0.01 and *ρ* = 0.05 are shown in Fig. 7, while the comparison with *ρ* = 0 can be found in Fig. 4(a2–c2).

**Figure 8:**
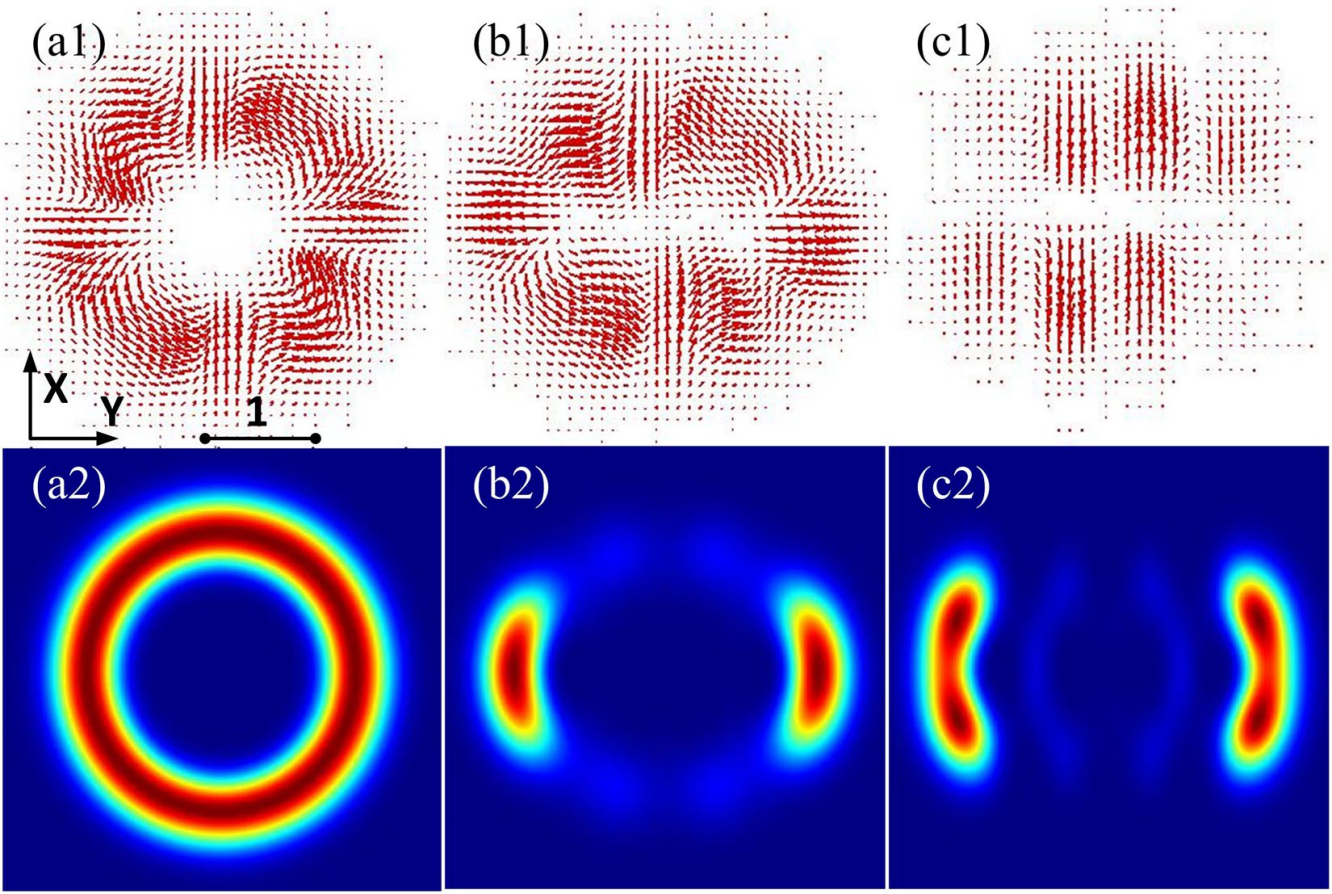
(**a1**–**c1**) Transverse energy flow (red arrows) of hIG wave packets in SNNM. (**a2**–**c2**) Angular momentum of hIG wave packets in SNNM. (**a1**–**a2**) *ε* = 10^−5^, (**b1**–**b2**) *ε* = 3, (**c1**–**c2**) *ε* = 10^5^. *P*_0_ = *P*_*c*_.

The local energy flow is usually expressed in terms of the Poynting vector. The Poynting vector has a magnitude of energy per unit area (or per unit time), and a direction which represents the energy flow at any point in the field. The vector potential is a linear polarization state. Given an *X* polarized vector potential **V** = *IG*^*h*^(*X*, *Y*, *Z*)*e*^*ikZ*^**e**_*X*_, where **e**_*X*_ is the unit vector along the *X* direction. The time-averaged Poynting vector can be expressed as $\langle {\bf{S}}\rangle =\frac{{{\bf{c}}}_{{\bf{0}}}}{4{\boldsymbol{\pi }}}\langle {\bf{E}}\times {\bf{B}}\rangle$^32^, where c_0_ is the velocity of light in a vacuum. Figures 8(a1–c1) show that the transverse energy flow appears to rotate counterclockwise around the vortex (distinct circulation of current). The length and direction of the arrows represent the intensity and direction of the Poynting vector respectively. As in mechanics, the time-averaged angular momentum density for the electromagnetic field is the angular momentum per unit area (per unit time), obtained by forming the cross product of the position vector with the time-averaged momentum density 〈**j**〉 = **r** × 〈**E** × **B**〉^33^. The spatial angular momentum distributions in Fig. 8(a2–c2) are more coherent than the intensity distributions in Fig. 5(a1–a3), and the positions of optical vortex may not be obvious.

## Conclusion

In summary, we have obtained a novel class of self-accelerating AiIG and AihIG optical breathers and solitons in SNNM from the method of separation of variables. The evolution properties of the AiIG and AihIG wave packets can be easily manipulated and modulated through adjusting *P*_0_/*P*_c_, the ellipticity *ε* and the strong nonlocality parameter *α*. The AiIG and AihIG optical solitons can be obtained when the input power equals the critical power, while the AiIG and AihIG breathers can be achieved with *P*_0_ < *P*_*c*_ or *P*_0_ > *P*_*c*_. The comparisons of analytical solutions with numerical simulations and numerical experiments of the AiIG and AihIG optical solitons show that the numerical results agree well with the analytical solutions in the case of strong nonlocality. For the first time, the spatial periodically oscillating (breather state) or steady (soliton state), temporal self-accelerating and approximately non-dispersion properties of AiIG and AihIG breathers and solitons are reported in SNNM, which is quite different from the case in free space^18^. If the last term of Eq. (1) in left part disappears, it becomes a free space condition, and the propagation of the spatial part is similar shown in Fig. 2(a1), which will expand due to the diffraction, and never show breathers and solitons.

In general, the analytical solution described here is applicable to other optical breathers and solitons such as self-accelerating AiHG optical breathers and solitons, self-accelerating AiLG optical breathers and solitons, and related self-accelerating Airy elegant breathers in SNNM. We foresee potential applications in signal processing due to the stabilized propagation properties of these wave packets.

## References

[1] Bandres MA, Gutiérrez-Vega JC (2004). Ince-Gaussian beams. Opt. Lett..

[2] Bandres MA, Gutiérrez-Vega JC (2005). Ince-Gaussian series representation of the two-dimensional fractional Fourier transform. Opt. Lett..

[3] Schwarz UT, Bandres MA, Gutiérrez-vega JC (2004). Observation of Ince-Gaussian modes in stable resonators. Opt. Lett..

[4] Schwarz UT, Bandres MA, Gutiérrez-vega JC (2005). Formation of Ince-Gaussian modes in a stable laser oscillator. Proc. SPIE.

[5] Deng D, Guo Q (2007). Ince-Gaussian solitons in strongly nonlocal nonlinear media. Opt. Lett..

[6] Deng D, Guo Q (2008). Propagation of Laguerre Gaussian beams in nonlocal nonlinear media. J. Opt. A: Pure Appl. Opt..

[7] Deng D, Guo Q (2011). Propagation of elliptic-Gaussian beams in strongly nonlocal nonlinear media. Phys. Rev. E.

[8] Conti C, Peccianti M, Assanto G (2004). Observation of optical spatial solitons in a highly nonlocal medium. Phys. Rev. Lett..

[9] Rotschild C, Cohen O, Manela O, Segev M, Carmon T (2005). Solitons in nonlinear media with an infinite range of nonlocality: first observation of coherent elliptic solitons and of vortex-ring solitons. Phys. Rev. Lett..

[10] Zhou GQ, Chen RP, Ru GY (2014). Propagation of an Airy beam in a strongly nonlocal nonlinear media. Laser Phys. Lett..

[11] Shen M, Gao J, Ge L (2015). Solitons shedding from Airy beams and bound states of breathing Airy solitons in nonlocal nonlinear media. Sci. Rep..

[12] Zhu W, Guan J, Deng F, Deng DM, Huang JW (2016). The propagation properties of the first-order and the second-order Airy vortex beams through strongly nonlocal nonlinear medium. Opt. Commun..

[13] Mihalache D (2012). Linear and nonlinear light bullets: recent theoretical and experimental studies. Rom. J. Phys..

[14] Chong A, Renninger WH, Christodoulides DN, Wise FW (2010). Airy-Bessel wave packets as versatile linear light bullets. Nat. Photon..

[15] Abdollahpour D, Suntsov S, Papazoglou DG, Tzortzakis S (2010). Spatiotemporal airy light bullets in the linear and nonlinear regimes. Phys. Rev. Lett..

[16] Zhong WP, Belić M, Zhang Y (2015). Three-dimensional localized Airy-Laguerre-Gaussian wave packets in free space. Opt. Express.

[17] Deng F, Deng D (2016). Three-dimensional localized Airy-Hermite-Gaussian and Airy-Helical-Hermite-Gaussian wave packets in free space. Opt. Express.

[18] Peng Y (2016). Self-accelerating Airy-Ince-Gaussian and Airy-Helical-Ince-Gaussian light bullets in free space. Opt. Express.

[19] Chen R, Dai C (2017). Three-dimensional vector solitons and their stabilities in a kerr medium with spatially inhomogeneous nonlinearity and transverse modulation. Nonlinear Dyn..

[20] Peng X (2017). Propagation properties of spatiotemporal chirped Airy Gaussian vortex wave packets in a quadratic index medium. Opt. Express.

[21] Peng X, Peng Y, Zhang L, Li D, Deng D (2017). Reversed Airy Gaussian and Airy Gaussian vortex light bullets in harmonic potential. Laser Phys. Lett..

[22] Berry MV, Balazs NL (1979). Nonspreading wave packets. Am. J. Phys..

[23] Ring JD, Howls CJ, Dennis MR (2013). Incomplete Airy beams: finite energy from a sharp spectral cutoff. Opt. Lett..

[24] Siviloglou GA, Christodoulides DN (2007). Accelerating finite energy Airy beams. Opt. Lett..

[25] Alonso MA, Bandres MA (2012). Spherical fields as nonparaxial accelerating waves. Opt. Lett..

[26] Siviloglou GA, Broky J, Dogariu A, Christodoulides DN (2008). Ballistic dynamics of Airy beams. Opt. Lett..

[27] Broky J, Siviloglou GA, Dogariu A, Christodoulides DN (2008). Self-healing properties of optical Airy beams. Opt. Express.

[28] Bandres MA, Rodríguez-Lara BM (2012). Nondiffracting Accelerating Waves: Weber waves and parabolic momentum. New J. Phys..

[29] Snyder AW, Mitchell DJ (1997). Accessible solitons. Science.

[30] Guo Q, Luo B, Yi F, Chi S, Xie Y (2004). Large phase shift of nonlocal optical spatial solitons. Phys. Rev. E.

[31] Deng D, Guo Q, Hu W (2009). Complex-variable-function Gaussian beam in strongly nonlocal nonlinear media. Phys. Rev. A.

[32] Deng D, Guo Q (2009). Airy complex variable function Gaussian beams. New. J. Phys..

[33] Sztul HI, Alfano RR (2008). The Poynting vector and angular momentum of Airy beams. Opt. Express.

